# Transcriptomic and Proteomic Profiling Reveal the Key Role of *AcMYB16* in the Response of *Pseudomonas syringae* pv. *actinidiae* in Kiwifruit

**DOI:** 10.3389/fpls.2021.756330

**Published:** 2021-11-11

**Authors:** Xiaojie Wang, Yawei Li, Yuanyuan Liu, Dongle Zhang, Min Ni, Bing Jia, Wei Heng, Zemin Fang, Li-wu Zhu, Pu Liu

**Affiliations:** ^1^School of Horticulture, Anhui Agricultural University, Hefei, China; ^2^School of Life Sciences, Anhui University, Hefei, China

**Keywords:** *Actinidia chinensis* var. *deliciosa*, bacterial canker, proteome, transcriptome, MYB TFs

## Abstract

Kiwifruit bacterial canker caused by *Pseudomonas syringae* pv. *actinidiae* (*Psa*), is an important disease of kiwifruit (*Actinidia* Lind.). Plant hormones may induce various secondary metabolites to resist pathogens via modulation of hormone-responsive transcription factors (TFs), as reported in past studies. In this study, we showed that JA accumulated in the susceptible cultivar *Actinidia chinensis* ‘Hongyang’ but decreased in the resistant cultivar of *A. chinensis* var. *deliciosa* ‘Jinkui’ in response to *Psa*. Integrated transcriptomic and proteomic analyses were carried out using the resistant cultivar ‘Jinkui’. A total of 5,045 differentially expressed genes (DEGs) and 1,681 differentially expressed proteins (DEPs) were identified after *Psa* infection. Two pathways, ‘plant hormone signal transduction’ and ‘phenylpropanoid biosynthesis,’ were activated at the protein and transcript levels. In addition, a total of 27 R2R3-MYB transcription factors (TFs) were involved in the response to *Psa* of ‘Jinkui,’ including the R2R3-MYB TF subgroup 4 gene *AcMYB16*, which was downregulated in ‘Jinkui’ but upregulated in ‘Hongyang.’ The promoter region of *AcMYB16* has a MeJA responsiveness *cis*-acting regulatory element (CRE). Transient expression of the *AcMYB16* gene in the leaves of ‘Jinkui’ induced *Psa* infection. Together, these data suggest that *AcMYB16* acts as a repressor to regulate the response of kiwifruit to *Psa* infection. Our work will help to unravel the processes of kiwifruit resistance to pathogens and will facilitate the development of varieties with resistance against bacterial pathogens.

## Introduction

Kiwifruit of the genus *Actinidia* Lind. is an economically important fresh fruit worldwide because of its rich nutritional value. Kiwifruit is a recently domesticated fruit crop with a short history of breeding, and its production is threatened by several emerging plant pathogens. Kiwifruit bacterial canker (KBC), caused by *Pseudomonas syringae* pv. *actinidiae* (*Psa*), has already become an economic threat to the kiwifruit industry worldwide (Tahir et al., 2019; Li et al., 2021). Many efforts have been made to uncover the population structure of *Psa*, and at least five types of populations, including biovars 1, 2, 3, 5, and 6, have been defined based on their virulence, toxin production, and host range, of which biovar 3 is responsible for the global pandemic (He et al., 2019). Most of the globally cultivated cultivars of kiwifruit, including *Actinidia chinensis* var. *chinensis*, *Actinidia chinensis* var. *deliciosa*, as well as accessions from *Actinidia arguta* and *Actinidia kolomikta*, are natural hosts of *Psa* (Tahir et al., 2019). Many cultivars of *A*. *chinensis* var. *chinensis*, including ‘Hongyang,’ ‘Hort16A,’ and ‘Jintao’, are considered more susceptible to biovar 3 than *A*. *chinensis* var. *deliciosa*, including ‘Jinkui’ and ‘Xuxiang’ (Li et al., 2020; Wang et al., 2020).

It has been reported that methyl jasmonate (MeJA), salicylic acid (SA), and abscisic acid (ABA) induce distinct biochemical and genetic responses during *Psa* colonization (Silva et al., 2021). These important plant hormones induce various secondary metabolites via modulation of hormone-responsive transcription factors (TFs) (Zhang et al., 2018). Transcriptional regulation of defense-related genes in plants is a crucial step in activating defense responses (Buscaill and Rivas, 2014). Among them, transcription factors play central roles in the regulation of transcription by forming a regulatory network in plants (Buscaill and Rivas, 2014). As a large TF gene family, MYB TFs are the most abundant and functional in plants and have attracted increasing attention due to their roles in plant development, metabolism and stress responses (Li et al., 2019). MYB proteins contain a highly conserved MYB DNA-binding domain (DBD) that comprises 1–4 imperfect MYB repeats. Each repeat covers 50–55 that fold into three α-helices, the second and third of which form a helix-turn-helix structure (Li et al., 2019; Jiang and Rao, 2020). Depending on the number of MYB domain repeats, this TF family can be divided into four categories, including 1R-, R2R3-, 3R-, and 4R-MYB proteins (Dubos et al., 2010). R2R3-MYBs are the most common type of MYB factor in land plants and have been classified into 23–90 subgroups in different studies (Li et al., 2019). Most MYB genes are positive regulators of transcription. For example, several MYB TFs act as positive regulators of pathogen defense. In *Arabidopsis thaliana*, *AtMYB30* is a positive regulator of the pathogen-induced hypersensitive response (Vailleau et al., 2002; Dubos et al., 2010); *AtMYB44* positively modulates resistance to the bacterial pathogen *P. syringae.* pv. *tomato* DC3000 (Zou et al., 2013). In apple, *MdMYB73* confers increased resistance to the fungal pathogen *Botryosphaeria dothidea* (Gu et al., 2021). In sweet cherry, overexpression of *PacMYBA* enhances resistance to DC3000 (Cui et al., 2018). *Arabidopsis* MYB96-mediated ABA signals enhance plant disease resistance by inducing SA biosynthesis (Seo and Park, 2010). In addition to positive regulation of plant disease resistance, negative regulation of R2R3-MYB has also been reported. For example, 22 MYBs to date have been reported as repressors to inhibit lignin and general phenylpropanoid synthesis in herbaceous and woody plants (Ma and Constabel, 2019). In *A. thaliana*, three MYB repressors regulating lignin or sinapate ester biosynthesis have been characterized, including *AtMYB3*, *AtMYB4*, and *AtMYB32* (Ma and Constabel, 2019). Within the MYB phylogeny, most MYB repressors belong to subgroup 4 of the R2R3-MYBs, which can be further separated into a general phenylpropanoid and lignin group and a flavonoid group (Yoshida et al., 2015). The subgroup 4 MYB TFs *FtMYB12*, *FtMYB14*, *FtMYB15* and *FtMYB16* in R2R3-MYB directly repress rutin biosynthesis in *Fagopyrum tataricum* (Ma and Constabel, 2019). Lignin is a major end product of the phenylpropanoid pathway and a key component of secondary cell walls and wood and plays important roles in mechanical strength and resistance to pathogens (Yoon et al., 2015). The phenylpropanoid pathway also produces resistance-related secondary metabolites, such as polyphenols and flavonoids (Yoon et al., 2015).

There are abundant MYB TF genes in the kiwifruit genome, and a total of 155 putative R2R3-type MYB TFs were identified from the kiwifruit genome sequence (Yu et al., 2019). Some of them, including *MYB7*, *MYB10*, *MYB110* and *AcMYB123*, have been reported for their role in the regulation of anthocyanin and proanthocyanin biosynthesis (Ampomah-Dwamena et al., 2019; Wang et al., 2019; Peng et al., 2020). Recently, it has also been reported that several MYB genes in kiwifruit are related to abiotic stress in plants (Wei et al., 2020). However, the response of the MYB genes in kiwifruit to *Psa* is not clear. Our study preliminarily elucidates the response of R2R3-MYB TF subgroup 4 gene *AcMYB16* in kiwifruit to *Psa* infection.

## Materials and Methods

### Plant Material and Pathogen Strains

One-year-old potted seedings *A. chinensis* var. *deliciosa* cultivar ‘Jinkui’ and the pandemic *Psa* strain JF8 (CCTCC AB2018305) were used to study the responses of kiwifruit to *Psa*. *S*train JF8 was originally isolated from *A. chinensis* var. *chinensis* cultivar ‘Jinfeng’ and has been characterized as belonging to biovar 3 (He et al., 2019). Plants were maintained in an aseptic room with 95% relative humidity, natural light and no further fertilization after being received from the nursery. For inoculation, the *Psa* strain was streaked on nutrient-sucrose agar (NSA) and incubated at 25°C for 48 h. Ten microliters of a bacterial suspension (1–2 × 10^7^ cfu/mL) prepared in sterile 0.85% w/v NaCl was inoculated in the plants chosen for investigation. The bacterial suspension was sprayed onto trees in their entirety. In parallel, control plants were treated in the same way with sterile 0.85% w/v NaCl solution. The inoculated and control plants were randomly distributed in the room at 15 ± 3°C. Leaves were sampled from the infected and control plants for further analyses after inoculation for 1 and 10 days. Each sample consisted of the leaves of one tree. Each group used three biological copies of trees.

### Abscisic Acid, Salicylic Acid, and Jasmonic Acid Analysis

One-gram leaf samples of *A. chinensis* var. *deliciosa* ‘Jinkui’ and *A. chinensis* var. *chinensis ‘*Hongyang’ at 1 day postinoculation (dpi) and 10 dpi of *Psa* were individually frozen and thoroughly ground into powder and extracted using isopropanol/hydrochloric acid buffer. After the addition of 20 mL of dichloromethane, vortexing and sonication for 10 min, the sample was centrifuged at 13,000 × *g* for 5 min. The substratum organic phase was dried with nitrogen and dissolved in 400 μl of methanol (with 0.1% formic acid) for LC-MS analysis. A poroshell 120 SB-C18 (2.1 × 150, 2.7 μm) was used with the following gradient elution program (solution A, methanol with 0.1% formic acid, and solution B, 0.1% formic acid): 0–2 min, 20% A; 4 min, 50% A; 10 min, 80% A; and 15 min, 20% A. Identification was carried out by comparing the references ABA, SA, and JA. The results are presented as mg/g FW. Three biological replicates were performed for each group.

### Protein Sample Preparation

One gram of *A. chinensis* var. *deliciosa* leaves at 1 dpi of *Psa* were thoroughly ground into powder in liquid nitrogen and extracted using ice-cold lysis buffer (7 M urea, 2 M thiourea, 4% CHAPS, 40 mM Tris-HCl, pH 8.5) containing 1 mM PMSF and 2 mM EDTA (final concentration). After 5 min, 10 mM DTT (final concentration) was added to the samples. The suspension was sonicated at 200 W for 15 min and then centrifuged at 4°C and 30,000 × *g* for 15 min. The supernatant was mixed well with a 5× volume of chilled acetone containing 10% (v/v) TCA and incubated at −20°C overnight. After centrifugation at 4°C and 30,000 × *g*, the supernatant was discarded. The precipitate was washed with chilled acetone three times. The pellet was air-dried and dissolved in lysis buffer (7 M urea, 2 M thiourea, 4% NP40, 20 mM Tris-HCl, pH 8.0–8.5). The suspension was sonicated at 200 W for 15 min and centrifuged at 4°C and 30,000 × *g* for 15 min. The supernatant was transferred to another tube. To reduce disulfide bonds in proteins of the supernatant, 10 mM DTT (final concentration) was added and incubated at 56°C for 1 h. Subsequently, 55 mM IAM (final concentration) was added to block the cysteines and incubated for 1 h in the darkroom. The supernatant was mixed well with a 5× volume of chilled acetone for 2 h at −20°C to precipitate proteins. After centrifugation at 4°C and 30,000 × *g*, the supernatant was discarded. The pellet was air-dried for 5 min and dissolved in 500 μL of 0.5 M Tetraethylammonium bromide (Applied Biosystems, Milan, Italy) and sonicated at 200 W for 15 min. Finally, samples were centrifuged at 4°C and 30,000 × *g* for 15 min. The supernatant was transferred to a new tube and quantified. The proteins in the supernatant were stored at −80°C until further analysis.

### Protein Digestion and iTRAQ Labeling

Protein solutions (100 μg) were diluted 4-times with 100 mM tetraethylammonium bromide, after which proteins were digested with Trypsin Gold (Promega, Madison, WI, United States) at 37°C (30:1, protein: trypsin) for 16-h. After trypsin digestion, peptides were dried by vacuum centrifugation. Peptides were reconstituted in 0.5 M TEAB and processed according to the manufacture’s protocol for 8-plex iTRAQ reagent (Applied Biosystems). Briefly, one unit of iTRAQ reagent was thawed and reconstituted in 24 μL isopropanol. Two leaves samples were labeled with iTRAQ tags 119 and 121, respectively. The peptides were labeled with the isobaric tags, incubated at room temperature for 2-h. The labeled peptide mixtures were then pooled and dried by vacuum centrifugation.

Peptides were separated using a Shimadzu LC-20AB HPLC Pump system coupled with a high-pH RP column. The iTRAQ-labeled peptide mixtures were reconstituted with 4 mL Buffer A (25 mM NaH_2_PO_4_ in 25% ACN, pH 2.7) and loaded onto a 4.6 × 250 mm Ultremex SCX column containing 5-μm particles (Phenomenex). The peptides were eluted at a flow rate of 1 mL/min with a gradient of buffer A for 10 min, 5-60% buffer B (25 mM NaH_2_PO_4_, 1 M KCl in 25% ACN, pH 2.7) for 27 min, 60–100% buffer B for 1 min. The system was then maintained at 100% buffer B for 1 min before equilibrating with buffer A for 10 min prior to the next injection. Elution was monitored by measuring the absorbance at 214 nm, and fractions were collected every 1 min. The eluted peptides were pooled into 20 fractions, desalted with a Strata X C18 column (Phenomenex) and vacuum-dried. IQuant software was used to quantify ITRAQ data (Wen et al., 2014).

### LC-ESI-MS/MS Analysis Based on Triple TOF 5600

Each fraction was re-suspended in buffer A (5% ACN, 0.1% FA) and centrifuged at 20,000 *g* for 10 min, the final concentration of peptide was about 0.5 μg/μL on average. 10 μL supernatant was loaded on a LC-20AD nanoHPLC (Shimadzu, Kyoto, Japan) by the autosampler onto a 2 cm C18 trap column. Then, the peptides were eluted onto a 10 cm analytical C_18_ column (inner diameter 75 μm) packed in-house. The samples were loaded at 8 μL/min for 4 min, then the 35 min gradient was run at 300 nL/min starting from 2 to 35% B (95% ACN, 0.1% FA), followed by 5 min linear gradient to 60%, then, followed by 2 min linear gradient to 80%, and maintenance at 80% B for 4 min, and finally return to 5% in 1 min.

Data acquisition was performed with a Triple TOF 5600 System (AB SCIEX, Concord, ON, Canada) fitted with a Nanospray III source (AB SCIEX, Concord, ON, Canada) and a pulled quartz tip as the emitter (New Objectives, Woburn, MA, United States). Data was acquired using an ion spray voltage of 2.5 kV, curtain gas of 30 psi, nebulizer gas of 15 psi, and an interface heater temperature of 150°C. The MS was operated with a RP of greater than or equal to 30,000 FWHM for TOF MS scans. For IDA, survey scans were acquired in 250 ms and as many as 30 product ion scans were collected if exceeding a threshold of 120 counts per second (counts/s) and with a 2+ to 5+ charge-state. Total cycle time was fixed to 3.3 s. Q2 transmission window was 100 Da for 100%. Four time bins were summed for each scan at a pulser frequency value of 11 kHz through monitoring of the 40 GHz multichannel TDC detector with four-anode channel detect ion. A sweeping collision energy setting of 35 ± 5 eV coupled with iTRAQ adjust rolling collision energy was applied to all precursor ions for collision-induced dissociation. Dynamic exclusion was set for 1/2 of peak width (15 s), and then the precursor was refreshed off the exclusion list.

### Protein Identification and Quantification Based on iTRAQ Data

Raw data files acquired from the Orbitrap were converted into MGF files using Proteome. Discoverer 1.2 (PD 1.2, Thermo) and the MGF file were searched. Proteins were identified and quantified using the Mascot 2.3.02 search engine (Matrix Science, London, United Kingdom) against Kiwifruit Genome Database (KGD) (Yue et al., 2020). The identified peptide sequences were then assembled into a set of accurately identified proteins based on the “simple principle”. To quantify proteins, peptides were automatically selected by calculating the reporter peak area using the default parameters of the Mascot software package. The resulting data set was auto-bias corrected, and the variations resulting from the unequal mixing of samples with different labels were eliminated. The Differentially abundant proteins (DAPs) between the *P. syringae.* pv. *actinidiae*-infected and CK samples were defined. To minimize the protein-level false-positive rates, a protein FDR of 1%, which was based on an established “picked” protein FDR strategy (Liu et al., 2018), was estimated after proteins were tentatively identified (protein-level FDR ≤ 0.01). All proteome data were deposited in the ProteomeXchange database under the accession PXD014787.

### Kiwifruit RNA Extraction and Sequencing

Total RNA was extracted from the leaves of *A. chinensis* var. *deliciosa* cultivar ‘Jinkui’ for subsequent RNA-Seq analysis. RNA was extracted from three biological replicates of frozen samples (100 mg) using the RNAprep Pure Plant Kit (Tiangen Biotech, Beijing, China). RNA quality was evaluated by a NanoPhotometer^®^ spectrophotometer (NanoDrop Technologies, Wilmington, DE, United States). RNA with an RNA integrity number > 8 according to the 2100 Bioanalyzer (Agilent, United States) was used to prepare cDNA libraries with the RNA Library Prep Kit (Illumina, San Diego, CA, United States). The resulting libraries were sequenced on a HiSeq 2000 platform (Illumina) to generate 100-bp paired-end reads. All RNA-Seq data were deposited in the National Center for Biotechnology Information (NCBI) database under the accession SRR16071936, SRR16071935, SRR16071934, SRR16071933, SRR16071932, and SRR16071931.

### Processing of Sequence Data and Mapping Reads to the Reference Genome

The sequenced data were filtered by removing adaptor sequences, empty reads, reads with more than 5% unknown nucleotides, low-quality sequences (base quality ≤ 20), or sequences with >10% Ns using SOAPnuke (version 1.5.2). Clean reads were mapped to the reference genome sequence using HISAT (version 0.1.6-beta). The reads were assembled into transcripts and compared with the reference gene of kiwifruit ‘Red 5’ (Liu et al., 2018) using Cufflinks. Gene expression was quantified using RSEM (RNA-Seq by Expectation Maximization, version 1.2.12). The data were normalized as fragments per kilobase of transcript per million fragments mapped (FPKM). The differences in transcript abundance between two genotypes were calculated based on the ratio of FPKM values. The FDR control method was used to identify the threshold of the *P*-value using Cuffdiff (included in the cufflinks package). Only transcripts with *P* ≤ 0.001 and |log2 (PT/CK)| > 1 were further analyzed. Cluster analysis of gene expression patterns was performed by Genesis based on the *K*-means method^1^. Gene ontology (GO) analysis was applied to predict gene function and to calculate the functional category distribution frequency. Principal component analysis (PCA) was performed using R tools 2.12.0 (Stacklies et al., 2007).

### Pathway Enrichment Analyses of Differentially Abundant Proteins and Differentially Expressed Genes

Functional annotations of the DAPs or DEGs were conducted using the Blast2GO program and the non-redundant protein database (NR; NCBI). Functional classification was performed based on the KEGG^2^ and COG databases^3^. *P* ≤ 0.05 was used to confirm the significance of the GO, KEGG pathway and MapMan analysis results.

### Quantitative Real-Time RT-PCR

Total RNA was reverse transcribed into first-strand cDNA using the M-MLV first strand kit (Invitrogen, United States) according to the manufacturer’s instructions. Twelve genes were chosen for confirmation by qRT-PCR with SYBR^®^GREEN Master Mix (Toyobo, Osaka, Japan). Primers for the chosen genes were designed with Primer Express software (Applied Biosystems, United States) and are presented in Supplementary Table 1. A qRT-PCR assay for gene expression analysis was performed on a Roche 480 Real-time PCR System (Roche Molecular Systems, Belleville, United States) using the ACTB gene as an endogenous control. Briefly, the primers for the target gene and ACTB were diluted in SYBR Mix, and 10 μL of the reaction mix was added to each well. The reactions were performed with an initial incubation at 50°C for 2 min and at 95°C for 1 min followed by 40 cycles of 95°C for 15 s, 60°C for 20 s and 72°C for 10 s. The levels of gene expression were analyzed with a LightCycler^®^ 480. Zero-template controls were included for each primer pair. Each PCR was carried out in triplicate, and the data are presented as the means ± SD. To assess the correlation between different platforms, Pearson correlations were calculated in SPSS 16.0 to compare the mRNA expression levels measured by RNA-Seq and qPCR.

### Sequence Analysis

The upstream 2-kb genomic DNA sequences of *Psa*-responsive AccR2R3-MYBs were submitted to the Plant CARE database^4^ to identify the *cis*-elements. A protein sequence similarity search was performed using BlastP. Multiple sequence alignment of MYB sequences was performed using Clustal Omega 8 (Sievers et al., 2011) and GeneDoc (Version 2.6.0.2) (Nicholas and Nicholas, 1997; Kumar et al., 2016). The phylogenetic trees were constructed using the MEGA 7 program (Sievers et al., 2011) based on the neighbor-joining method with 1000 bootstrap replicates.

### Transient Expression of AcMYB16 in ‘Hongyang’ Leaves

Sequences of AcMYB16 were PCR amplified from cDNA of the *A. chinensis* var. *deliciosa* cultivar ‘Jinkui’ and cloned into vector pCAMBIA1300. Gene-specific primers (AcMYB16-F, 5′-GAGAACACGGGGGACTCTAGAATGGGGAGATCACCGAG-3′; AcMYB16-R, 5′-GCCCTTGCTCACCATGGATCCCATTTC TGGAAAATCTTTCAG-3′) were used for AcMYB3R amplification, with underlined nucleotides as restriction enzyme cutting sites. The overexpression vector pCAMBIA1300 driving the expression of the candidate gene was transformed into *Agrobacterium tumefaciens* strain GV3101 by electroporation (Peng et al., 2019). Leaves of ‘Hongyang’ were infiltrated with a mixture of *Psa* and empty vector and or a mixture of *Psa* and *Agrobacterium* (1:1) and kept under the same growth conditions. Leaves were photographed and harvested at 14 days after infiltration. Leaves at the junction of disinfected spots (100 mg) were surface-disinfected via immersion in 75% alcohol for 1 min and 15% sodium hypochlorite solution for 1 min and rinsed five times with sterile water. The samples were ground by adding 300 μL of sterile water and diluted to 10^–3^. Each sample was repeated three times in KB medium and cultured at 20°C for 2 days. The number of *Psa* was observed and recorded by electron microscopy.

### Statistical Analysis

All statistical analyses in this study were conducted using the Statistical Program SPSS 13.0 for Windows (SPSS Inc., Chicago, IL, United States). Analysis of variance (ANOVA) was performed, and Duncan’s multiple range test was used for mean separation. The statistical significance in this experiment was all applied at the level *P* < 0.05.

## Results

### Differential Accumulation of Abscisic Acid, Salicylic Acid, and Jasmonic Acid in Two Kiwifruit Cultivars After *Psa* Inoculation

**FIGURE 1:**
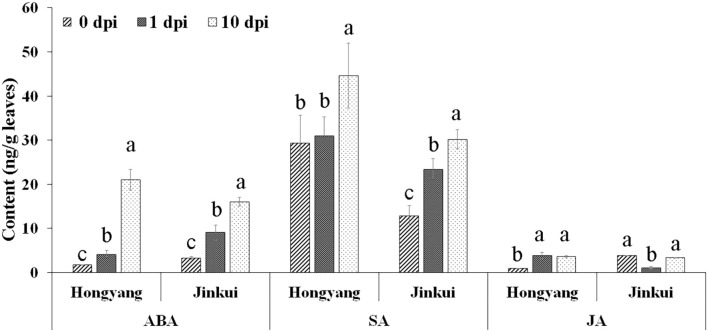
Hormone concentration analysis of abscisic acid (ABA), salicylic acid (SA), and jasmonic acid (JA) at 0, 1, and 10 dpi of *Psa* in ‘Jinkui’ and ‘Hongyang’ leaves. Values are the means ± SE (*n* = 3). Different letters indicate significant differences at a *p*-value < 0.05.

Abscisic acid, SA, and JA hormones were determined at different time points (0, 1, and 10 days) after *Psa* inoculation in two kiwifruit cultivars. The results showed that ABA and SA were increased in both cultivars after *Psa* inoculation at different times, but JA accumulated in ‘Hongyang’ and decreased in ‘Jingkui’ (Figure 1) at 1 dpi. The JA content of Jingkui recovered to the level before *Psa* inoculation at 10 dpi. This result suggests that the two varieties may have different response pathways to *Psa*.

### Transcriptome and Proteome Profiles of *A. chinensis* var. *deliciosa* After Inoculation With *Psa*

To reveal the global picture of gene responses to KBC, *P. syringae.* pv. *actinidiae* strain JF8 was used to treat the resistant *A. chinensis* var. *deliciosa* cultivar ‘Jinkui,’ and a transcriptomic analysis was performed. The RNA-Seq generated an average of 11.47 Gigabytes (GB) of clean data (*Q*_30_ > 95.96%). A total of 91,120,152–126,564,610 clean reads were obtained. Approximately 62.54% of reads mapped to the kiwifruit reference genome *A*. *chinensis* var. *chinensis* ‘Red 5’ (Wei et al., 2020). In total, 5045 differentially expressed genes (DEGs, |log_2_ fold change| > 1, *P* < 0.05) were identified, including 1,538 upregulated and 3,507 downregulated genes (Supplementary Table 2). Among them, twelve random DEGs were chosen for qRT-PCR (quantitative real-time PCR) verification. Correlations between the expression patterns of these genes in RNA-Seq and qRT-PCR were evaluated using SPSS 16.0. As shown in Supplementary Figure 1, a high correlation was obtained between the two methods, with a correlation coefficient of *R*_2_ = 0.9353.

Protein profile analysis produced 33,995 peptide spectra from the kiwifruit leaf libraries, resulting in 8,067 proteins (Supplementary Table 3). PCA revealed observed variability differences between the treatment and control groups (Supplementary Figure 2). By comparing the multiple changes in expression between the treatment and control groups, correlation analysis was performed at the transcriptome and proteome levels. Correlations were revealed between mRNAs and their coding proteins (*R* = 0.687, Supplementary Figure 3).

### Comparison of Gene Ontology and KEGG Pathway Enrichment of *A. chinensis* var. *deliciosa* After Inoculation With *Psa* at the Transcript and Protein Levels

Gene ontology functional classification and KEGG pathway enrichment analysis were performed on those DEGs. According to the GO functional analysis, 49 GO terms were significantly enriched, especially “cellular process,” “metabolic process,” “membrane,” “cell,” “binding,” and “catalytic activity” (Supplementary Figure 4A). These enriched functional processes were correlated with the symptoms of BCK, including dark brown spots surrounded by yellowish halos on leaves as well as the presence of dark red exudates on canes and trunks. Following KEGG enrichment analysis, “plant–pathogen interaction,” “ABC transporters,” “phenylpropanoid biosynthesis,” “plant hormone signal transduction,” “glycosphingolipid biosynthesis – lacto and neolacto series,” “alpha-linolenic acid metabolism,” “starch and sucrose metabolism,” and “biosynthesis of secondary metabolites” were enriched for the most significant pathways (Supplementary Figure 4B).

**FIGURE 2:**
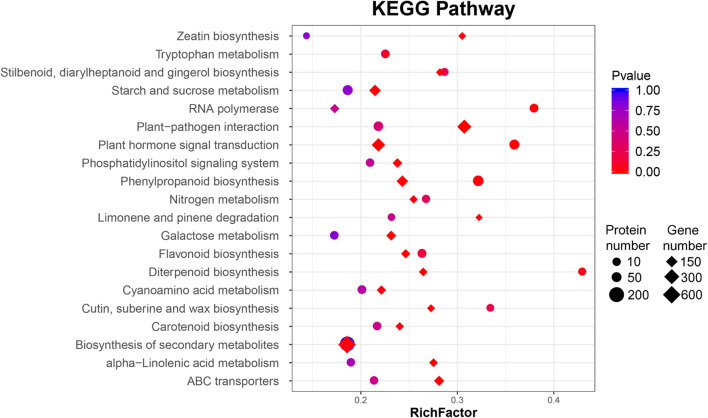
Bubble diagrams of the KEGG enrichment of differentially expressed genes (DEGs) and proteins (DAPs) of ‘Jinkui’ leaves at 1 dpi of *Psa*.

In these interesting pathways, the number of DEPs was significantly smaller than that of DEGs. According to the GO functional analysis, 51 GO terms were significantly enriched, including “catalytic activity,” “binding,” “cell,” “cellular process,” and “metabolic process” (Supplementary Figure 5A). Most genes were differentially expressed in the transcriptome analysis, but there was no significant difference in protein level. Only two DEP pathways, “plant hormone signal transduction” and, “phenylpropanoid biosynthesis,” had statistical significance consistent with the DEGs (Figure 2, Supplementary Table 4, and Supplementary Figure 5B).

### Hormone Signaling Pathway Enrichment

**FIGURE 3:**
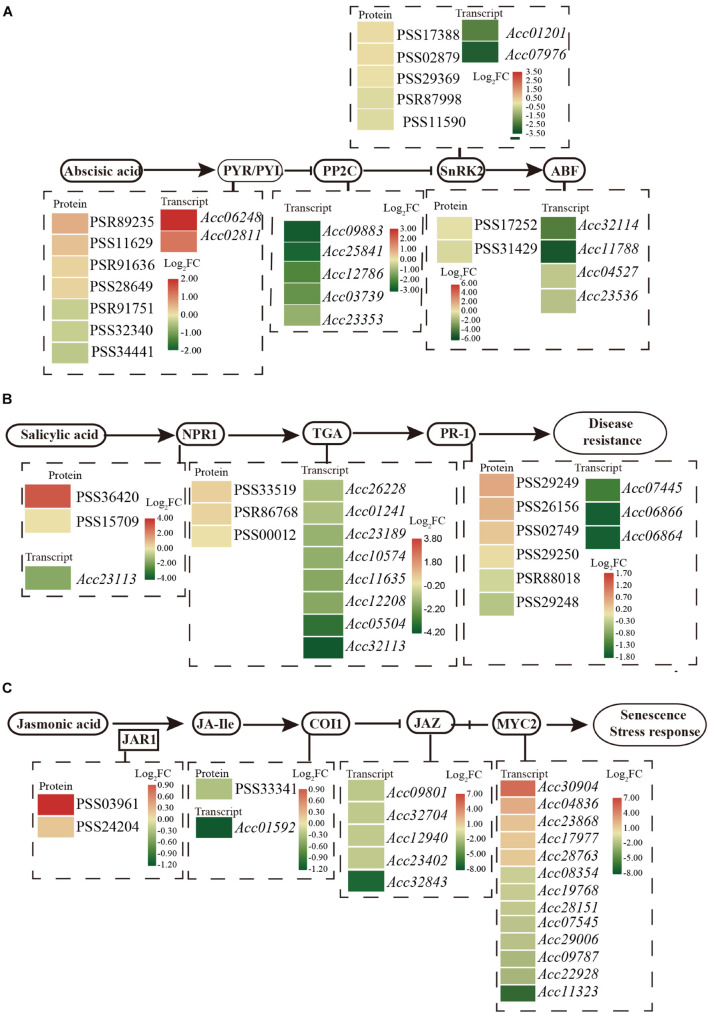
Schematic of the transcriptome and proteome revealed the regulation of ABA **(A)**, SA **(B)**, and JA **(C)** in the plant hormone signal transduction pathway at 1 dpi of *Psa* in ‘Jinkui’ leaves.

Phytohormone networks consisting of SA, JA, and ethylene signaling are required for plant pathogen-associated molecular pattern (PAMP)-triggered immunity (PTI) and effector-triggered immunity (ETI). After *Psa* inoculation, 34 proteins and 263 genes were enriched with the pathway “plant hormone signal transduction” by KEGG analysis (Supplementary Table 4). These included numerous proteins/transcripts involved in ABA, SA, and JA (Figure 3).

Abscisic acid perception and signal transduction depend on its binding to receptors of the pyrabactin resistance1/PYR1-like/regulatory components, the subsequent inhibition of clade A type 2C protein phosphatases (PP2Cs), and the phosphorylation of ion channels and transcription factors by protein kinases of the SnRK2 family. A total of 13 genes and 14 proteins were detected for the ABA signaling response to *Psa* infection. Two PYR/PYL (Pyrabactin Resistance1/PYR-Like) genes (Acc06248 and Acc02811) were found to be significantly upregulated, and four proteins had also accumulated. Correspondingly, five genes encoding PP2C (protein phosphatase 2C, *Acc09883*, *Acc25841*, *Acc12786*, *Acc03739*, and *Acc23353*), a negative regulator of ABA signal transduction, were downregulated at the transcript level. Moreover, the expression levels of two genes that encoded SnRK2 (serine/threonine-protein kinase SRK2, *Acc01201* and *Acc07976*) were downregulated, and four genes that encoded ABF (ABA responsive element binding factor, *Acc32114*, *Acc11788*, *Acc04527*, and *Acc23536*) were also downregulated by *Psa* infection (Figure 3A). These results are consistent with ABA accumulation in ‘Jinkui’ leaves after *Psa* inoculation.

The NPR-1 (non-expressor of PR gene) and PR1 (pathogenesis-related protein-1) genes, which are used as markers for SA-mediated disease resistance, accumulated at the protein level but were downregulated at the transcript level. Eight annotated genes encoding the TGA transcription factor were all downregulated, but three proteins accumulated, which exerted their activity by binding to the core *cis*-elements (TGACGs) of target genes, including *PR-1* and *NPR1* (Figure 3B). Our results showed that proteins in the SA-related pathways of ‘Jinkui’ accumulated after *Psa* inoculation. This is consistent with the accumulation of SA in ‘Jinkui’ leaves, indicating that SA has a positive response to *Psa*.

For JA, two proteins of jasmonic acid-amino synthetase (JAR1, EC: 6.3.2.52) were upregulated, but coronatine-insensitive protein 1 (COI1) was downregulated at the transcript and protein levels, indicating that JA-isoleucine (JA-Ile) may be cumulative. Five jasmonate ZIM domain-containing protein (JAZ) genes and eight transcription factor MYC2 genes were downregulated, and five transcription factor MYC2 genes were upregulated at the transcript level. Among them, the genes *Acc30904* (log_2_FC = 5.30) and *Acc04836* (log_2_FC = 2.5) may be important positive regulation genes in the ‘Jinkui’ response to *Psa* infection (Figure 3C).

### Phenylpropanoid Pathway Enrichment

**FIGURE 4:**
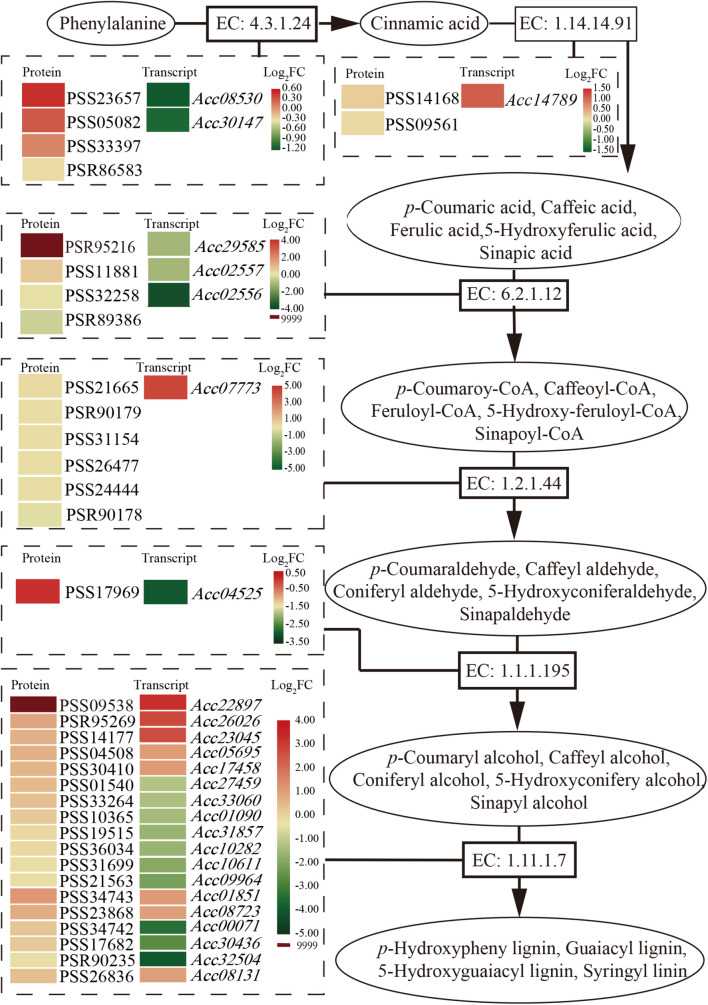
Schematic of the transcriptome and proteome revealing the regulation of phenylpropanoid biosynthesis at 1 dpi of *Psa* in ‘Jinkui’ leaves.

Phenylpropanoids usually perform their function by inducible antimicrobial compounds, as well as signal molecules such as SA, in plant-microbe interactions. In total, 149 genes and 49 proteins were enriched that participate in multiple branches of the phenylpropanoid pathway (Supplementary Table 4). Six major enzymes related to the phenylpropanoid biosynthesis pathway were mainly promoted at the protein level (Figure 4). The first common step of phenylpropanoid biosynthesis is catalyzed by the enzyme EC: 4.3.1.24 (phenylalanine ammonia-lyase, PAL). Although two genes encoding PAL showed downregulated expression at the transcript level, three proteins were promoted. One gene and two proteins of the enzyme EC: 1.14.14.91 (*trans*-cinnamate 4-monooxygenase, CA4H), and one gene and five proteins of the enzyme EC: 1.2.1.44 (cinnamoyl-CoA reductase) were upregulated and promoted. The PSR95216 protein of the enzyme EC: 6.2.1.12 (4-coumarate-CoA ligase, 4CL) and the PSS09538 protein of the enzyme (peroxidase, POD) were highly expressed (log_2_FC = 9999) after *Psa* inoculation. The results may suggest that phenolic compound accumulation and lignin content increased in the phenylpropanoid biosynthesis pathway after *Psa* inoculation in ‘Jinkui.’ Lignin is a major end product of phenylpropanoid pathway and is involved in plant disease resistance.

### Transcription of R2R3-MYB Genes in Two Kiwifruit Cultivars After *Psa* Inoculation

**FIGURE 5:**
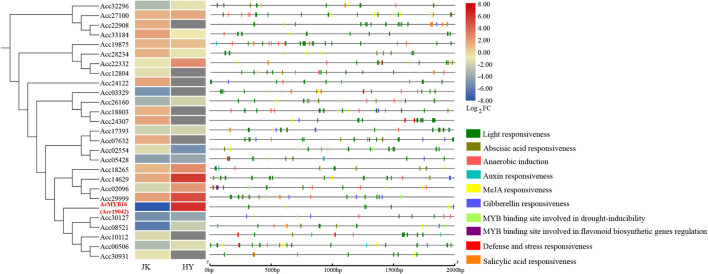
Distribution of *cis*-acting regulatory elements (CREs) involved in the responsiveness of the promoters of 27 candidate R2R3-MYBs.

After *Psa* inoculation, 27 of the R2R3-MYB TFs in *A. chinensis* var. *deliciosa* ‘Jinkui’ were differentially expressed, 12 of which were upregulated and 15 of which were downregulated. These 27 genes were analyzed by qRT-PCR in *A. chinensis* var. *chinensis ‘*Hongyang’, 16 of them were significantly changed, 6 of them were upregulated and 10 of them were downregulated (Figure 5). To establish the “regulation–expression” cue of these candidate AccR2R3-MYBs, the *cis*-acting regulatory elements (CREs) located 2,000 bp upstream of the 27 genes were identified using the PlantCARE database. The distribution of CREs involved in responsiveness and development is shown in Figure 5. The hormone-related CREs mainly included “Abscisic acid responsiveness,” “Auxin responsiveness,” “MeJA responsiveness,” “Gibberellin responsiveness,” and “Salicylic acid responsiveness.” Among the 27 genes, the most special gene of AcMYB16 (*Acc19042*) was downregulated (log_2_-fold change = −7.9) in *A. chinensis* var. *deliciosa* ‘Jinkui’ and upregulated (log_2_-fold change = 6.1) in *A. chinensis* var. *chinensis* ‘Hongyang’ in response to *Psa*. The promoter region of AcMYB16 have five light responsiveness CREs and one MYB binding site involved in drought-inducibility CRE, suggesting that this gene may be involved in light and drought response. Interestingly, there is also one MeJA responsiveness CRE in the promoter region of *AcMYB16*, indicating that *AcMYB16* might be a susceptible gene involved in *Psa* infection in kiwifruit by responding to JA. We hypothesized that *AcMYB16* might be a negative R2R3-MYB TF gene in response to *Psa* related to JA.

### Protein Sequence Analysis of AcMYB16

The protein sequences of AcMYB16 shared the highest identity (70.34%) with AtMYB16 (AT5G15310) in the *A. thaliana* genome. After clustering AcMYB16 with 8 subgroup 4 TFs from *A. thaliana* (Zhang et al., 2018), *F. tataricum* (Zhang et al., 2018), and *Chrysanthemum morifolium* (Zhu et al., 2013), a phylogenetic tree was constructed that confirmed AcMYB16 classified into subgroup 4 of the R2R3-MYB TFs (Supplementary Figure 6). The results show that AcMYB16 may be a MYB16 protein. Comparison of amino acid sequences between AcMYB16 and MYB subgroup 4 proteins revealed that they share highly conserved R2R3 domains in the N-terminal regions but are more divergent at the C-terminal ends (Supplementary Figure 7). These typical motifs of MYB subgroup 4, including C2 and C4 motifs (Zhu et al., 2013), were not detected at the C-terminus of AcMYB16. In addition, compared with FtMYB 16 and FtMYB 13, EAR motif and SID domain related to response JA are not conserved in AcMYB16 (Zhou et al., 2017; Zhang et al., 2018). These results suggest that AcMYB16 is a novel MYB16 protein.

### MYB Induced *Psa* Infection of Kiwifruit Leaves

**FIGURE 6:**
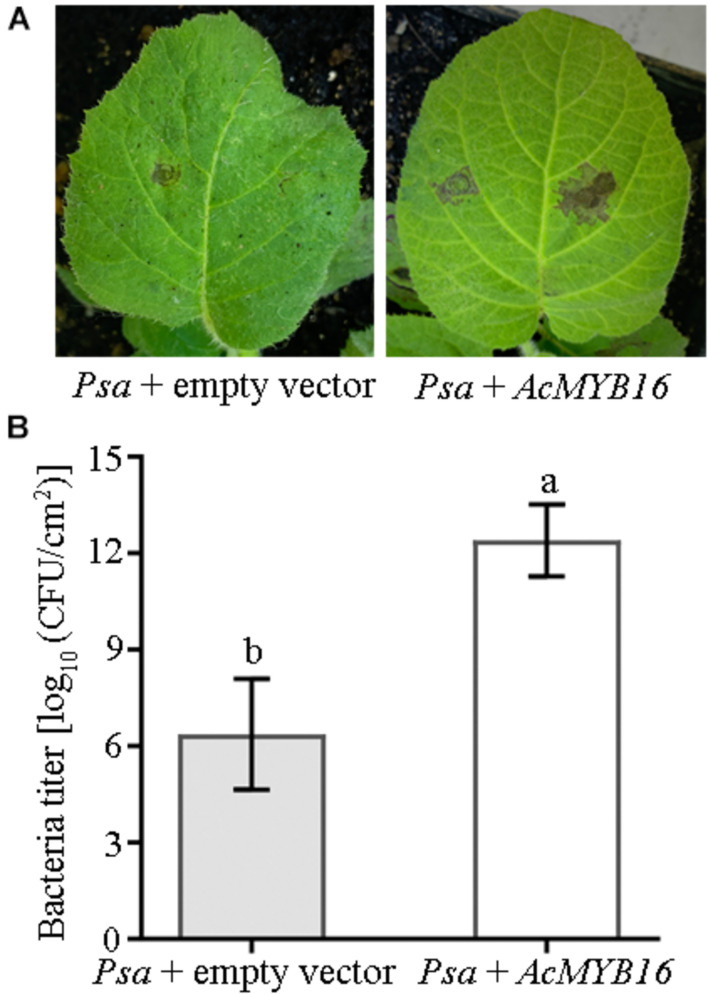
Transient expression of *AcMYB16* in ‘Jinkui’ leaves induced *Psa* infection **(A)** and enhanced plant sensitivity to *Psa* **(B)**.

Transient expression of the *AcMYB16* gene in the leaves of ‘Hongyang’ induced *Psa* infection (Figure 6A). ‘Hongyang’ leaves with transient expression of the *AcMYB16* gene were more susceptible to disease. The bacterial concentration of leaves with *AcMYB16* transient expression inoculated with *Psa* was twice as high as that of the vector control leaves inoculated with *Psa* only (Figure 6B). The results indicate that *AcMYB16* is a gene capable of inducing *Psa* infection in leaves.

## Discussion

In plants, SA, ABA, and JA and signaling pathways are critical for plant resistance against *P. syringae* (Flors et al., 2018). In this study, SA and ABA were significantly increased after *Psa* inoculation in *A. chinensis* var. *deliciosa* ‘Jinkui’ (Figure 1) with the accumulation of related proteins (Figure 3) after inoculation with *Psa*. The change of SA and ABA content in *A. chinensis* var. *chinensis* ‘Hongyang’ was consistent with that in ‘Jinkui.’ The accumulation of SA is central to the full manifestation of PTI and ETI at and near infection sites (Flors et al., 2018; Klessig et al., 2018). In previous studies, proteomic and transcriptomic analyses were performed to analyze the defense response of kiwifruit to *P. syringae.* pv. *actinidiae* infection (Wang et al., 2018). It has been demonstrated that elicitors of the SA pathway limit disease severity in both *A. chin*e*nsis* var. *chinensis* and *deliciosa*. The genes PR1, PR8, ICS, and PAL were elicited after infection by *P. syringae.* pv. *actinidiae* in *A. chinensis* var. *deliciosa.* Comparison of the transcriptomes of *A. chinensis* var. *chinensis*, *A. arguta* and *Actinidia eriantha* led to the suggestion that resistance to *P. syringae.* pv. *actinidiae* was related to the expression of a number of long non-coding RNAs that act in concert with coding genes (Wang et al., 2017). In *Arabidopsis*, the phytohormones ABA and JA mediate suppression of MAP kinases (MAPKs) (Mine et al., 2017), which are exploited by DC3000 via production of T3SS effectors, such as AvrPtoB, AvrB, HopAM1, and HopX1, as well as the phytotoxin coronatine (Geng et al., 2012). In this study, JA showed different changes in the two varieties of ‘Jinkui’ and ‘Hongyang’ after inoculation with *Psa* (Figure 1). JA is an important plant hormone that induces the biosynthesis of various secondary metabolites, such as phenylpropanoid and flavonoids, via the modulation of JA-responsive TFs (Geng et al., 2012). A number of MYB TFs have been found to interact with members of the jasmonate ZIM domain protein family (Zhang et al., 2018).

MYB TFs are widely distributed in higher plants, are the most abundant and powerful transcription factors in the transcription factor family, and play a central role in plant defense resistance, but their functions in the kiwifruit defense response to *Psa* infection remain incompletely understood (Zhang et al., 2018). A total of 155 putative R2R3-MYB TFs were identified from the *A. chinensis* ‘Red 5’ genome sequence (Yue et al., 2020), 27 of which were involved in the response to *Psa*. The R2R3-MYB TF gene *AcMYB16* was downregulated in the resistant cultivar *A. chinensis* var. *deliciosa* ‘Jinkui’ but upregulated in the susceptible cultivar *A. chinensis* var. *Chinensis ‘*Hongyang*’* in response to *Psa* at 1 dpi. Analysis of the promoter region of *AcMYB16* found that this gene has a MeJA-responsive CRE. Hormone assays also showed that JA accumulated in ‘Hongyang’ and decreased in ‘Jingkui’ at 1 dpi of *Psa* (Figure 1). The results showed that *AcMYB16* acts as a negative regulatory gene in response to JA in kiwifruit infected with *Psa*. Furthermore, protein sequence analysis results showed that AcMYB16 is a protein classified into subgroup 4 of the R2R3-MYB TFs.

**FIGURE 7:**
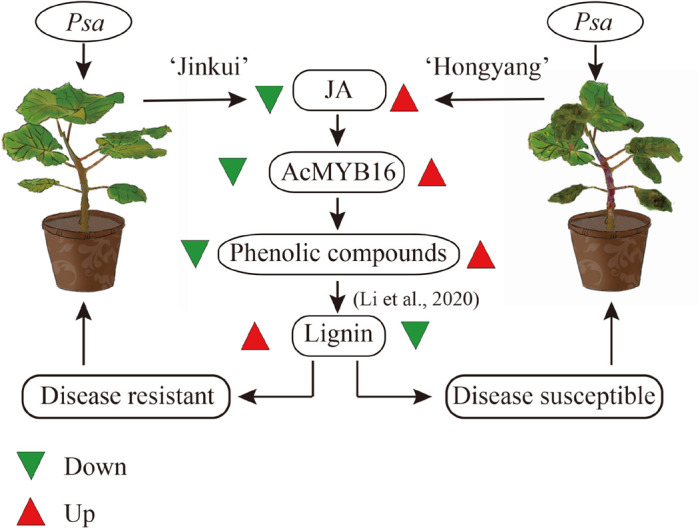
Possible scheme for metabolism following *Psa* attack in ‘Jinkui’ and ‘Hongyang’ leaves.

In *Arabidopsis*, several R2R3-MYB TFs, such as AtMYB4, AtMYB7, and AtMYB32, belong to subgroup 4, which act as transcriptional repressors owing to the EAR motif (Dubos et al., 2010). JA signaling results in the activation of TFs, which regulate gene expression through specific binding to *cis*-acting elements in the promoters of target genes (Zhou and Memelink, 2016). In *F. tataricum*, JA-responsive FtMYB16 specifically acts as a phenylpropanoid biosynthesis repressor, dependent on the EAR motif, while FtMYB13 acts as a repressor, dependent on the conserved Asp residues (FtMYB13^D285N^) of the SID domain (Zhou et al., 2017; Zhang et al., 2018). In *Populus*, MYB189 is a transcriptional repressor, and the amino acid GDDYGNHGMIKKE at the C-terminal end is required for its repressive activity (Zhang et al., 2018; Jiao et al., 2019). However, in this study, sequence alignment showed that AcMYB16 does not have any of these conditions (Figure 7).

As one of the model plant pathogens, previous studies of DC3000 in *Arabidopsis* and tomato showed its capacity for immunity suppression and JA manipulation (Xin et al., 2018). The woody-infecting pathogen has evolved a system enabling the breakdown of woody tissue of plant trees (Nowell et al., 2016; Xin et al., 2018). In plants, physical defense by the cell wall is one of the most powerful mechanisms used by plants to defend themselves against pathogens. The cell wall is a recalcitrant network of polysaccharides, including cellulose, hemicellulose (such as xyloglucan and arabinoxylan), lignin and pectin. Upon pathogen attack, plants often deposit callose-rich cell wall appositions (i.e., papillae) at sites of attempted pathogen penetration, accumulate phenolic compounds and various toxins in the wall and synthesize lignin-like polymers to reinforce the wall (Zhao and Dixon, 2014). In our study, a total of 149 genes and 49 proteins were enriched that participate in multiple branches of the phenylpropanoid pathway after *Psa* inoculation (Supplementary Table 4). These data showed that suppression of cell wall synthesis is crucial for *Psa* infection. However, our previous study showed that most of the phenolic compounds were reduced in ‘Jinkui’ but increased in ‘Hongyang’ after *Psa* inoculation, while lignin changed in the opposite direction (Li et al., 2020), indicating that phenolic compounds were negatively related to kiwifruit *Psa* resistance, and lignin was positively related to kiwifruit *Psa* resistance. It has been reported that various MYB transcription factor genes, including a large class of plant transcription factors, directly or indirectly regulate the expression of lignin biosynthesis genes in many plant species (Kim et al., 2020). For example, the biosynthesis of syringyl lignin is dependent on MYB103 in Arabidopsis stems (Öhman et al., 2013). Heterologous expression of AtMYB61 in *Oryza sativa* increased lignin content mainly by enriching syringyl units as well as *p*-coumarate and tricin moieties in the lignin (Koshiba et al., 2017). MYB156 and MYB189 act as repressors of lignin in *Populus tomentosa* (Koshiba et al., 2017; Yang et al., 2017). Overexpression of *PtoMYB156* in *P. tomentosa* repressed phenylpropanoid biosynthetic genes, leading to reductions in the amounts of total phenolics and lignin. In contrast, knockout of PtoMYB156 in poplar resulted in the ectopic deposition of lignin (Yang et al., 2017). In ‘Jinkui,’ phenolic compounds did not accumulate, although there was a significant accumulation of proteins related to phenolic synthesis. The accumulation of proteins associated with lignin synthesis and the increase in lignin content suggest that phenolic substances may be used to synthesize lignin to resist *Psa* accompanied by downregulation of the R2R3-MYB TF subgroup 4 gene *AcMYB16*. However, in ‘Hongyang’, the accumulation of phenolic compounds led to the proliferation of bacteria and a decrease in lignin (Li et al., 2020) accompanied by upregulation of the TF gene *AcMYB16*, which made ‘Hongyang’ vulnerable (Figure 7).

## Conclusion

In this study, we showed that SA, ABA and JA were involved in the response to *Psa* in the resistant cultivar *A. chinensis* var. *deliciosa* ‘Jinkui’ and in the susceptible cultivar *A*. *chinensis* var. *chinensis* ‘Hongyang.’ JA accumulated in ‘Hongyang’ and decreased in ‘Jingkui.’ We further determined the responses of the resistant cultivar ‘Jinkui’ at the gene and protein levels, which indicated that *Psa* infection activates “plant hormone signal transduction” and “phenylpropanoid biosynthesis.” In addition, a total of 27 R2R3-MYB TFs were involved in the response to *Psa* of ‘Jinkui,’ including the R2R3-MYB TF subgroup 4 gene *AcMYB16*, which was downregulated in the resistant cultivar ‘Jinkui’ but upregulated in ‘Hongyang’ at 1 dpi. The promoter region of *AcMYB16* has a MeJA-responsive CRE. Transient expression revealed that *AcMYB16* could increase *Psa* infection in kiwifruit leaves. Our results showed that *AcMYB16* acts as a repressor gene to regulate the biosynthesis of lignin in response to JA in kiwifruit infected with *Psa*.

## Data Availability Statement

The datasets presented in this study can be found in online repositories. The names of the repository/repositories and accession number(s) can be found below: Proteomic data was deposited in ProteomeXchange under accession no: PXD014787; Sequencing data was deposited in NCBI SRA BioProject under accession no: PRJNA766305.

## Author Contributions

XW and YaL designed and performed the whole experiments and wrote the manuscript. YuL, DZ, MN, and BJ performed data analysis. WH and ZF provided scientific suggestion. PL and L-wZ supervised. All authors contributed to the article and approved the submitted version.

## Conflict of Interest

The authors declare that the research was conducted in the absence of any commercial or financial relationships that could be construed as a potential conflict of interest.

## Publisher’s Note

All claims expressed in this article are solely those of the authors and do not necessarily represent those of their affiliated organizations, or those of the publisher, the editors and the reviewers. Any product that may be evaluated in this article, or claim that may be made by its manufacturer, is not guaranteed or endorsed by the publisher.

## Footnotes

1
http://genome.tugraz.at/

2
http://www.genome.jp/kegg/

3
http://www.ncbi.nlm.nih.gov/COG/

4
http://bioinformatics.psb.ugent.be/webtools/plantcare/html/

## Abbreviations

4CL: 4-coumarate-CoA ligase
ABA: abscisic acid
CA4H: *trans*-cinnamate 4-monooxygenase
DBD: DNA-binding domain
DAPs: differentially abundant proteins
Dpi: day postinoculation
COI1: coronatine-insensitive protein 1
CREs: *cis*-acting regulatory elements
JA: jasmonic acid
JA-Ile: JA-isoleucine
JAR1: jasmonic acid-amino synthetase
JAZ: jasmonate ZIM domain-containing protein
KGD: kiwifruit Genome Database
KBC: kiwifruit bacterial canker
MAPKs: MAP kinases
MeJA: methyl jasmonate
NSA: nutrient-sucrose agar
MAPKs: MAP kinases
POD: peroxidase
*Psa*: *Pseudomonas syringae* pv. *actinidiae*
SA: salicylic acid
TFs: transcription factors.
