# Supplementary material for: Transcriptomic and Proteomic Profiling Reveal the Key Role of AcMYB16 in the Response of Pseudomonas syringae pv. actinidiae in Kiwifruit
Source: Front Plant Sci. 2021 Nov 11;12:756330. doi: 10.3389/fpls.2021.756330 (PMC8632638; doi:10.3389/fpls.2021.756330)
Supplement: Supplementary file 1 [file Data_Sheet_1.doc]

**Supplementary Material**


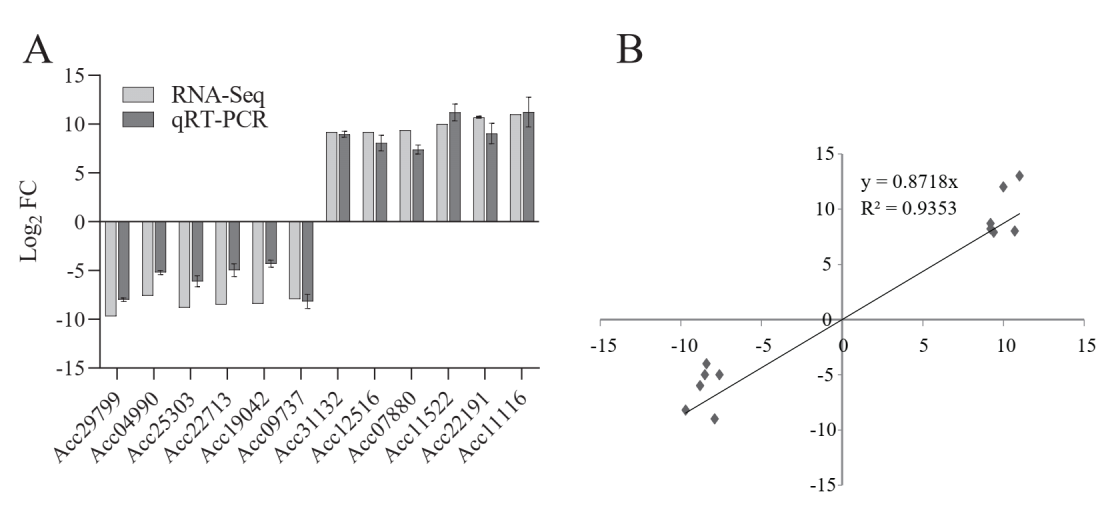


**Figure S1 Validation of the transcriptome data. (A) The results from RNA-seq analysis were compared with those from qPCR. Bars represent the mean ± SD (n=3). (B) Comparison and log2 expression of 12 selected differentially regulated genes from infected kiwifruit as measured by RNA-Seq and qPCR.** Positive and negative log_2_ expression ratios represent up- and downregulation, respectively, compared with uninfected kiwifruit.


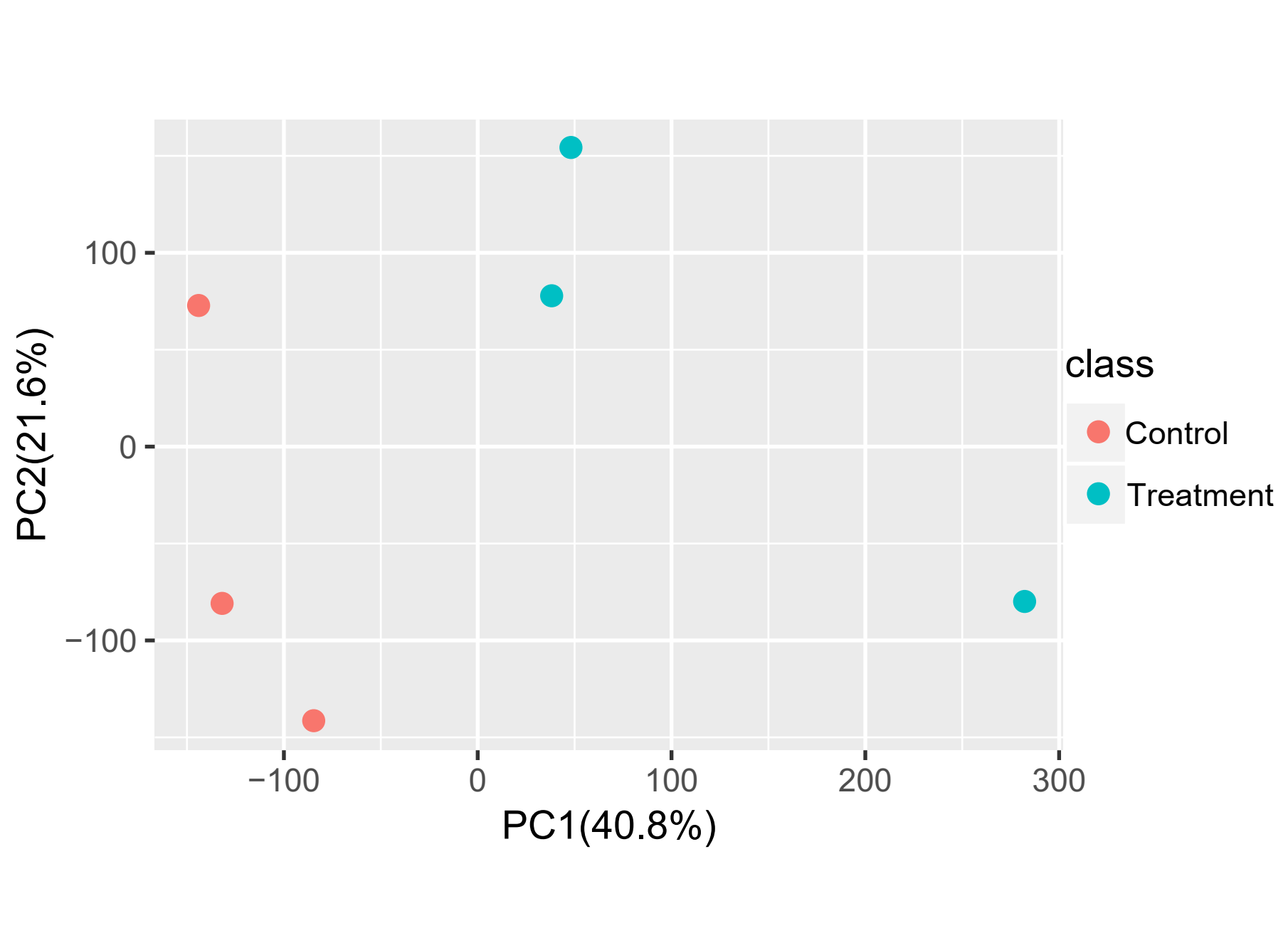


**Figure S2 Principal component analysis (PCA) of metabolite and gene expression data.** ‘Jinkui’ leaves were sampled from the control and infected plants after inoculation at 1 dpi.

**
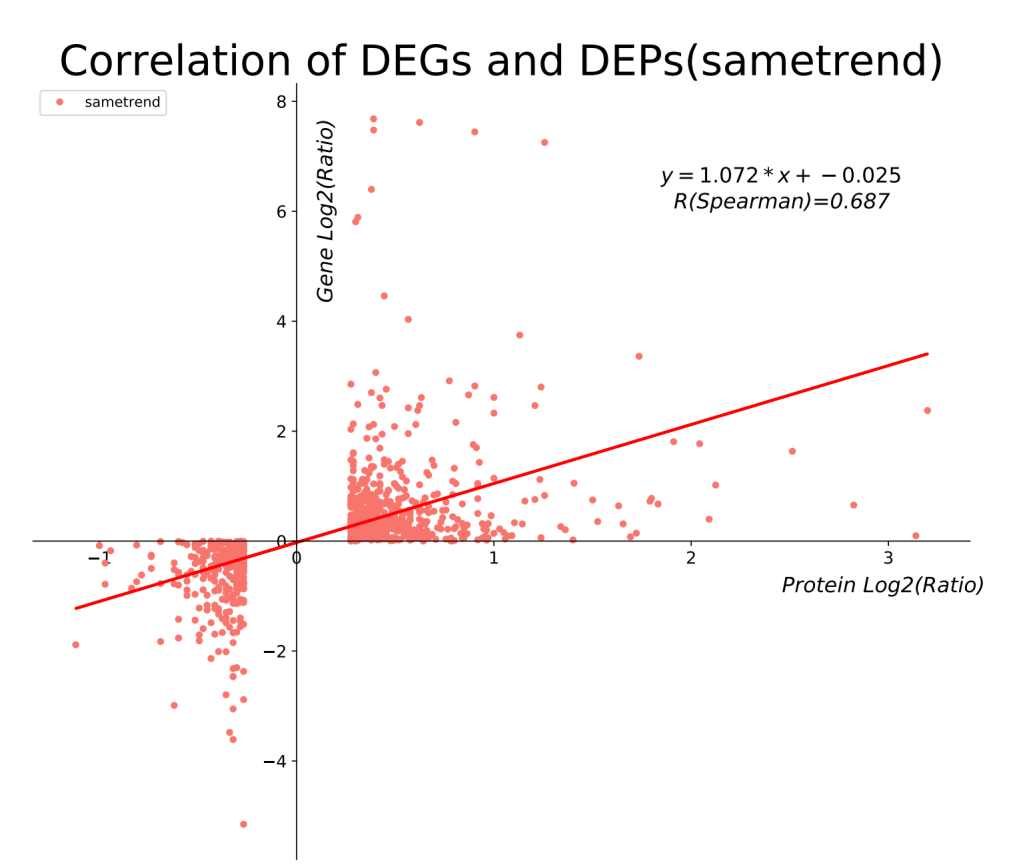
**

**Figure S3 Correlation analysis performed at the transcriptome and proteome levels.** According to the expression trend of protein level and mRNA level, the correlation between mRNA with the same change trend and protein was analyzed.

**
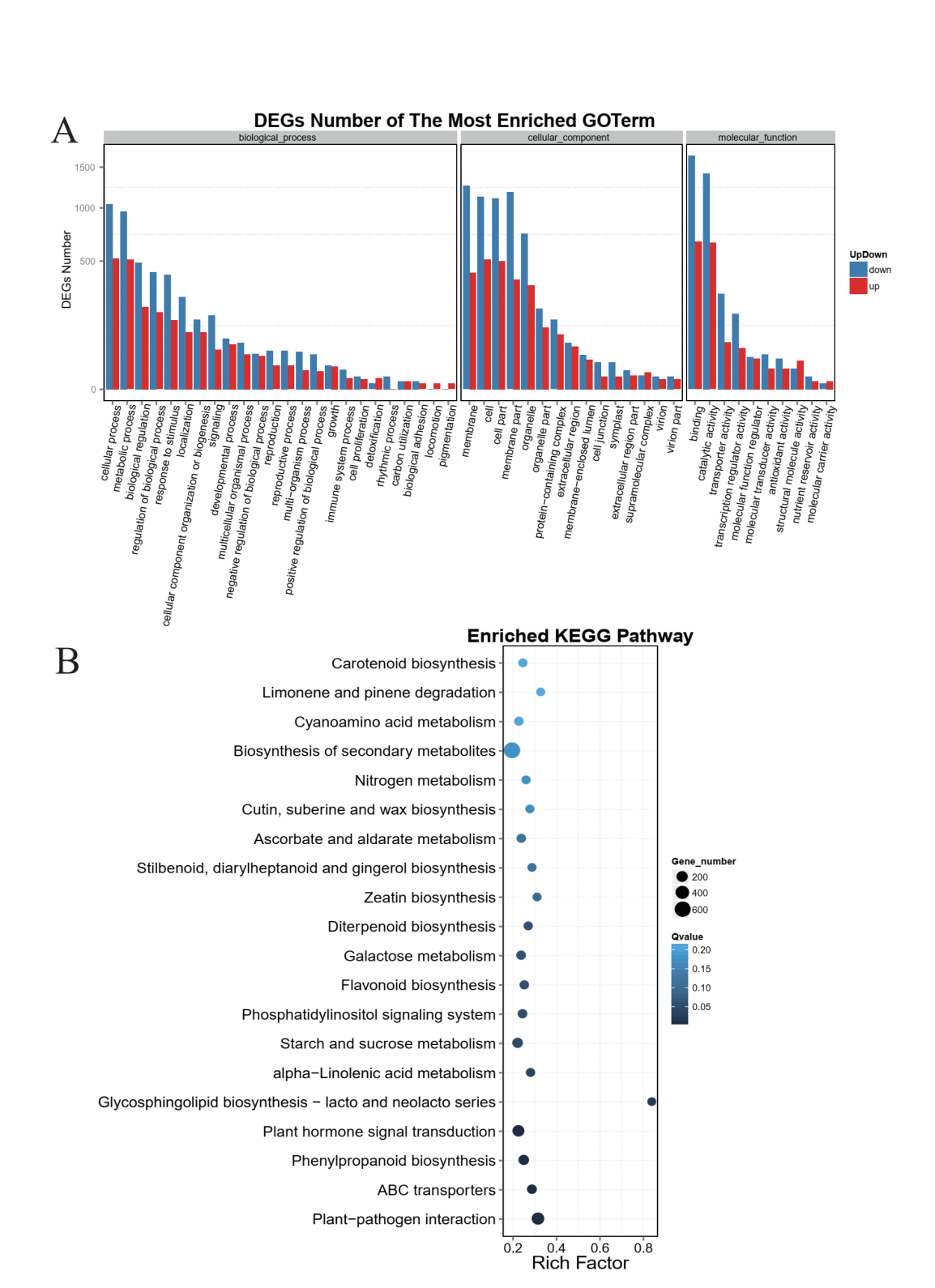
**

**Figure S4 GO (A) and KEGG (B) enrichment of differentially expressed genes (DEGs) of ‘Jinkui’ leaves at 1 dpi of *Psa*.**


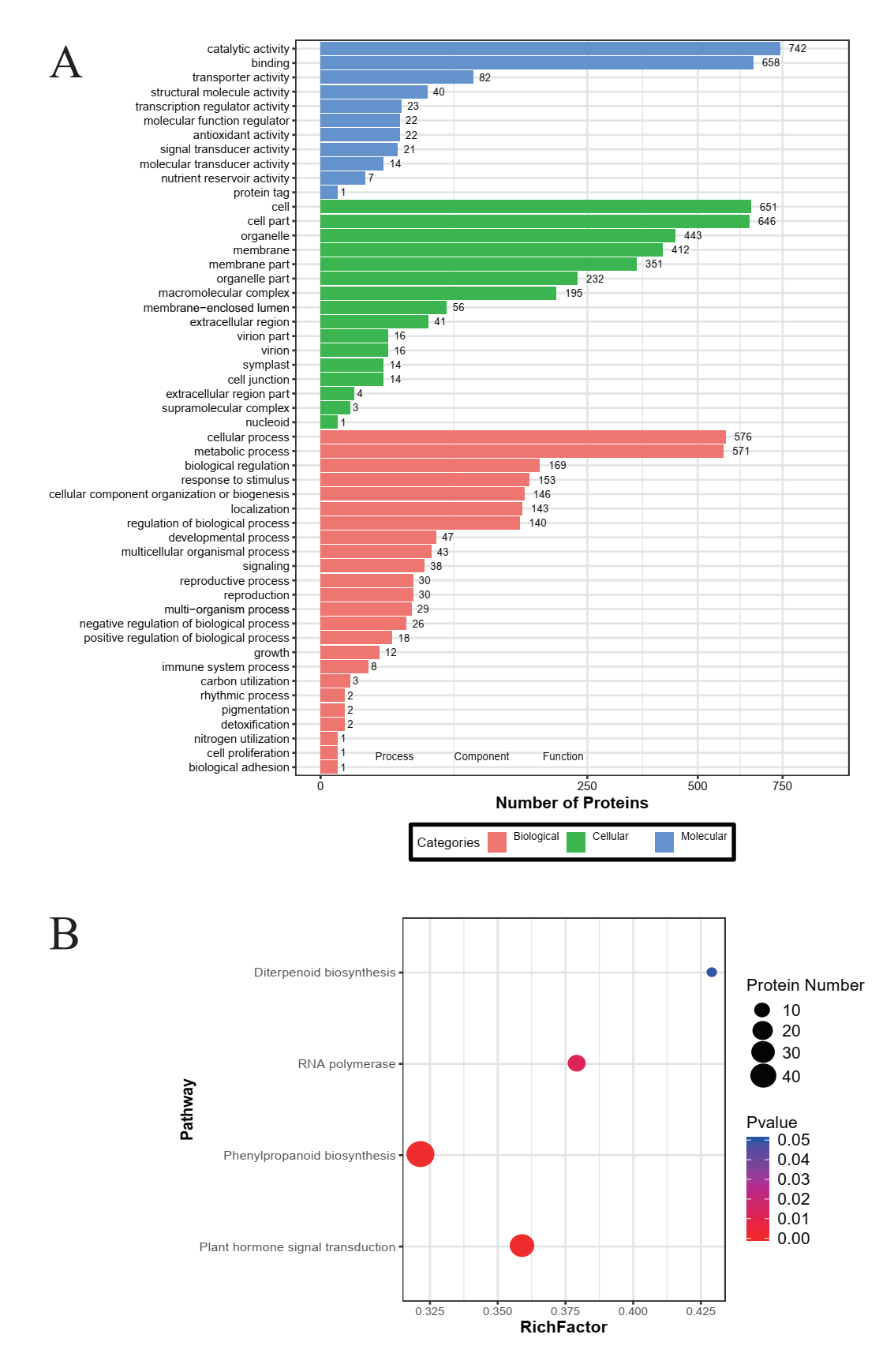


**Figure S5 GO (A) and KEGG (B) enrichment of differentially expressed proteins (DAPs) in ‘Jinkui’ leaves at 1 dpi of *Psa*.**

**
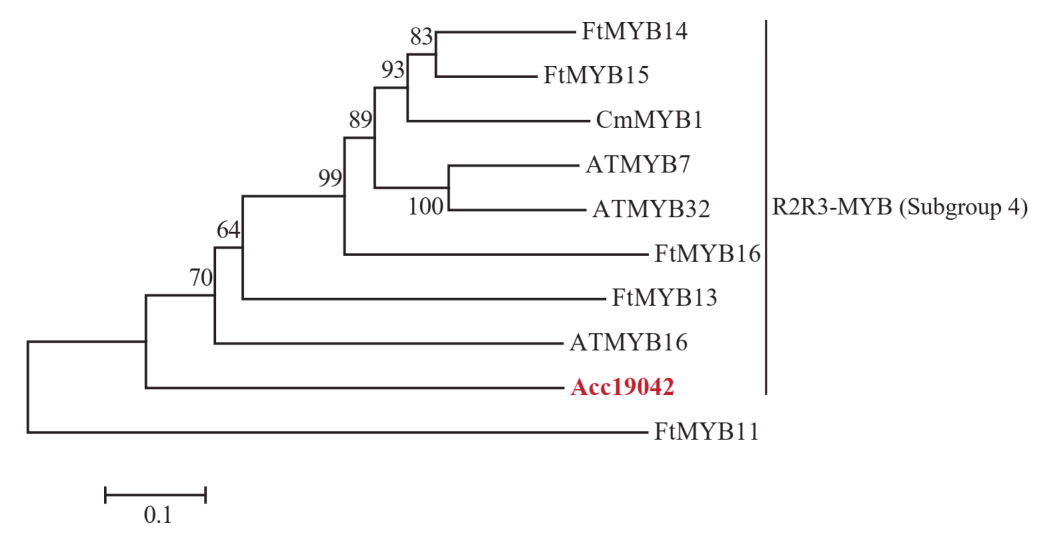
**

**Figure S6 Phylogeny of R2R3-MYB TFs from subgroup 4 R2R3-MYB TFs from *Arabidopsis*, *F. tataricum*, and *C. morifolium*. The phylogenetic tree is based on the alignment of complete protein sequences.**

**
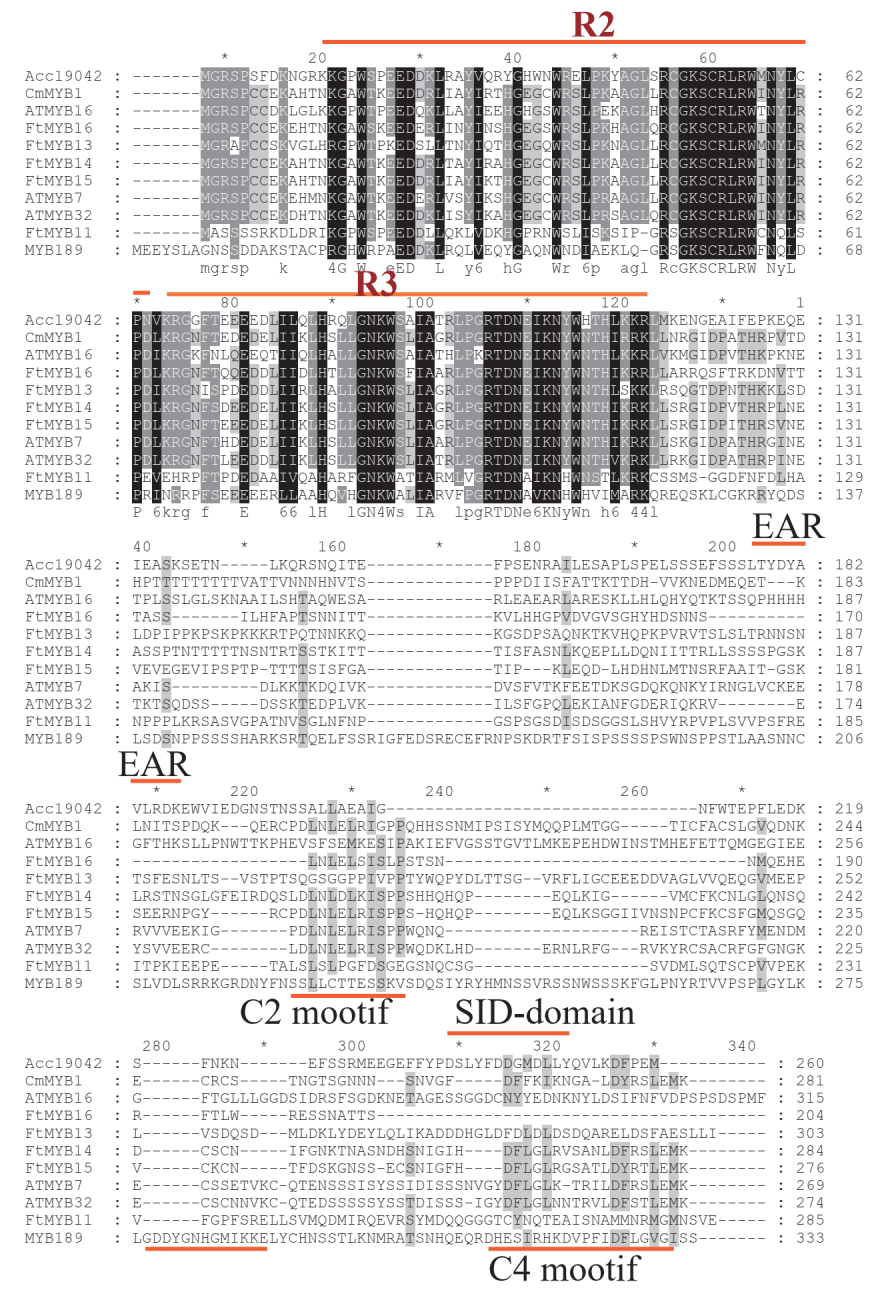
**

**Figure S7 Amino acid sequence alignment of a clade of subgroup 4 R2R3-MYB TFs. Sequences were aligned using Clustal W and MEGA version 7.**

**Table S1 Sequences of primers used in qRT-PCR analysis.**

**Table S2 Differentially expressed genes (DEGs, |log_2_ fold change| > 1, *P* < 0.05) were identified in** ***A. c.* var. *deliciosa* cultivar ‘Jinkui’ after inoculation with *Psa*.**

**Table S3** **Protein profile analysis produced 8,067 proteins from the kiwifruit leaf libraries.**

**Table S4 Pathways of differential accumulation of proteins and differential expression of related transcripts in *A. c. var. deliciosa* cultivar ‘Jinkui’ after *Psa* inoculation.**
